# Causes of early postpartum complications that result in visits to the emergency department

**DOI:** 10.1371/journal.pone.0260101

**Published:** 2021-11-29

**Authors:** Prabhpreet Hundal, Rahim Valani, Cassandra Quan, Shayan Assaie-Ardakany, Tanmay Sharma, Maher Abou-Seido, Leila Salehi, Qamar Amin, Simina Luca

**Affiliations:** 1 Department of Obstetrics & Gynecology, University of Alberta, Edmonton, Alberta, Canada; 2 Faculty of Medicine, University of Toronto, Toronto, Ontario, Canada; 3 Faculty of Medicine, McMaster University, Hamilton, Ontario, Canada; 4 University of Illinois at Chicago, Chicago, Illinois, United States of America; 5 William Osler Health System, Brampton, Ontario, Canada; Ohio State University Wexner Medical Center Department of Surgery, UNITED STATES

## Abstract

**Objective:**

This study aimed to review the reasons why postpartum women present to the emergency department (ED) over a short term (≤10 days post-delivery) and to identify the risk factors associated with early visits to the ED.

**Methods:**

This retrospective chart review included all women who delivered at a regional health system (William Osler Health System, WOHS) in 2018 and presented to the WOHS ED within 10 days after delivery. Baseline descriptive statistics were used to examine the patient demographics and identify the timing of the postpartum visit. Univariate tests were used to identify significant predictors for admission. A multivariate model was developed based on backward selection from these significant factors to identify admission predictors.

**Results:**

There were 381 visits identified, and the average age of the patients was 31.22 years (SD: 4.83), with median gravidity of 2 (IQR: 1–3). Most patients delivered via spontaneous vaginal delivery (53.0%). The median time of presentation to the ED was 5.0 days, with the following most common reasons: abdominal pain (21.5%), wound-related issues (12.6%), and urinary issues (9.7%). Delivery during the weekend (OR 1.91, 95% CI 1.00–3.65, P = 0.05) was predictive of admission while *Group B Streptococcus* positive patients were less likely to be admitted (OR 0.22, CI 0.05–0.97, P<0.05)

**Conclusions:**

This was the first study in a busy community setting that examined ED visits over a short postpartum period. Patient education on pain management and wound care can reduce the rate of early postpartum ED visits.

## Introduction

Canada has over 370,000 births each year and providing emergency care during the postpartum period remains a priority [1]. After delivery, mothers are discharged after an average of 2.0 days for vaginal births and after 3.4 days for cesarean sections [2]. The emergency department (ED) is frequently attended for care prior to the scheduled postpartum visit due to ease of access.

The postpartum period is defined as six weeks following delivery, while most visits to the ED occur within 7–10 days [3,4]. Prior studies have looked at postpartum ED visits but none of them focused on the very early period [4,5]. Taking care of a newborn, undergoing postpartum anatomical and physiological changes, as well as the social and financial issues all require the new mother’s attention. This is a critical time for emotional bonding with the baby and visiting the ED is an additional stressor.

This study aimed to characterize postpartum ED visits within 10 days of delivery, the reason for their visit, and whether they required obstetrical consultation. We also sought to identify predictors for patients who presented to the ED and were admitted.

## Methods

We conducted a retrospective chart review of women who delivered at William Osler Health System (WOHS), which is a regional health care system. Patients who delivered between January 1, 2018 and December 31, 2018 and who presented to the WOHS Emergency Department within 10 days of delivery were included. This study was approved by the WOHS Research Ethics Board (18–0068). The WOHS is composed of the following three hospitals located in the Greater Toronto Area: Peel Memorial Centre, Brampton Civic Hospital, and Etobicoke General Hospital. In 2018, 7878 deliveries occurred at the above-mentioned locations. This study was approved by WOHS Research Ethics Board (18–0068), who waived the requirement for consent given the nature of the study.

All live-born deliveries between January 1, 2018 and December 31, 2018 at WOHS were included. Patients were identified through a request to the Clinical Decision Support Unit for all deliveries that took place during the time period and presented to the emergency department within 10 days. Patients were excluded if they had a prolonged postpartum hospital stay (greater than 24 hours for a vaginal delivery and 48 hours for a Cesarean delivery), as these patients likely experienced other complications that required prolonged stay. We included those who presented to any of the three WOHS EDs within 10 days of delivery.

For each delivery, information on patient (age, gravidity-parity status, pre-existing comorbidities) and delivery characteristics (date and gestational age at delivery, Group B streptococcus (GBS) status, type of delivery, duration of rupture of membranes, any complications at delivery) were collected by manual extraction from the chart. For each ED visit, information about the visit (date, number of postpartum days, presenting complaint, discharge diagnosis, treatment, disposition, consultations) was extracted. These variables were chosen based on a blended model of the literature search results and by the authors *a priori*.

All statistical analyses were performed using IBM SPSS Version 25 statistical package. Descriptive analysis was carried out to assess the distribution of traits within the study sample. The effects of maternal age, gravidity, parity, gestational age, duration of rupture of membrane, GBS status, day of delivery (i.e., weekend vs. weekday), and type of delivery (i.e., C-section vs. vaginal) on admission status were assessed via univariate analysis. Missing data were imputed using the multiple imputation technique and pooled estimates were used for analysis. A multivariate model was then developed based on backward selection from the set of significant factors identified on univariate analysis. All retention criteria were set at P<0.05.

## Results

There were 429 unique visits during the period and 381 were included in the final analysis and the remainder not included as they met the exclusion criteria. The average age of the patients was 31.22 years (range: 19–43 years, SD 4.83), and the average gestational age at delivery was 38.78 weeks (SD: 2.29).

Most deliveries were identified as spontaneous vaginal deliveries (53.0%), and an additional 7.9% were assisted vaginal deliveries. Weekend deliveries occurred in 26.2% of cases. The majority of patients were GBS-negative (71.9%). On univariate analysis, weekend deliveries and positive GBS status were significantly associated with admission (P<0.05). In the multivariable analysis, weekend delivery (OR 1.91, 95% CI 1.00–3.65, P = 0.05) was associated with admission, while a positive GBS status was less likely to be admitted (OR 0.22, 95% CI 0.05–0.97, P<0.05). See Table 1.

**Table 1:** Baseline patient characteristics of patients and predictors of admission.

Variable	Mean (SD) ^a^ Median (IQR) ^b^ % of cohort ^c^	Univariate analysis p-value	Multivariate analysis Odds Ratio	Multivariate analysis 95% CI	p-value
**Patient Characteristics**					
Age^a^	31.22 years (4.83)	0.605			
Gravidity^a^	2.28 (1.47)	0.741			
Parity^a^	0.72 (0.86)	0.151			
Gestational age at delivery^b^	38.78 weeks (2.29)	0.229			
**Peri-partum factors**					
Duration of rupture of membranes^b^	5 hrs (2–11)	0.426			
Group B Strep status (positive)	67%	0.039	0.22	0.05–0.97	0.05
Vaginal delivery	60.9%	0.412			
Cesarean Section delivery	39.1%	0.997			
Weekend delivery	26.2%	0.037	1.91	1.00–3.65	<0.05

Table 2 provides a summary of the medical history, type of delivery, complications during delivery, and GBS status. The most common medical conditions were previous gynecological conditions (17.1%), endocrine issues (12.3%), and a prior C-section (10.0%).

**Table 2:** Frequency and percentage of key patient characteristics.

		Frequency/count	Percent of total
**Medical History**	Mental health (depression, anxiety, bipolar disorder, panic)	21	5.5
	Hypertension	11	2.9
	Cardiac/pulmonary	23	6.0
	Previous Cesarean section	38	10.0
	Complications in previous pregnancies	36	9.4
	Gynecological (incl. surgery)	65	17.1
	Other abdominal surgeries	20	5.2
	Breast (incl. surgery)	5	1.3
	Urinary tract	9	2.4
	Gastrointestinal/liver	9	2.4
	Neurological	12	3.1
	Endocrine	47	12.3
	MSK/rheum	2	0.5
	Hematological	19	5.0
	Thromboembolic disease	3	0.8
	Others	3	0.8
	None	191	50.1
**Type of delivery**	Spontaneous vaginal delivery	202	53.0
	Cesarean section	149	39.1
	Vacuum-assisted vaginal delivery	22	5.8
	Forceps-assisted vaginal delivery	8	2.1
**Complications at delivery**	Cord around neck or body; knot	79	20.7
	Shoulder dystocia	6	1.6
	Post-partum hemorrhage	15	3.9
	Stillbirth/neonatal death	5	1.3
	Manual removal of placenta	2	0.5
	None	280	73.5
**GBS**	Negative	274	71.9
	Positive	67	17.6
	Unknown	40	10.5
**Number of births on weekends**		100	26.2
**Consultations**	Obstetrician	98	25.7
	Anesthesia	6	1.6
	Anesthesia, internal medicine	1	0.3
	Cardiology	1	0.3
	Neurology	1	0.3
	General surgery	3	0.8
	Intensive Care Unit	1	0.3
	Infectious disease	3	0.8
	Internal medicine	15	3.9
	Interventional radiology	1	0.3
	Urology	1	0.3
**Disposition**	Home or transfer	335	87.9
	Admitted	46	12.1

Only one- quarter of the patients required an obstetric consultation when they presented to the ED, and most were then discharged (87.9%). Table 3 provides the discharge diagnosis and relative frequency. The most common diagnoses were pain (21.5%) and wound-related issues (12.6%).

**Table 3:** Frequency and percentage of discharge diagnoses.

	TOTAL COHORT		ADMITTED PATIENTS	
Discharge diagnosis	Frequency	Percent	Frequency	Percent
Breast problems	6	1.6	1	2.2
Cardiovascular/Respiratory issues	19	5.0	0	0
Edema/swelling	12	3.1	0	0
Endometritis	29	7.6	11	23.9
Gastrointestinal issues	21	5.5	0	0
Hypertension/preeclampsia	32	8.4	8	17.4
Left without being seen	5	1.3	0	0
Mental health issues	2	0.5	0	0
Normal	11	2.9	0	0
Others	18	4.7	1	2.2
Pain	82	21.5	6	13.0
Post-epidural issues	7	1.8	1	2.2
Postpartum hemorrhage	5	1.3	4	8.7
Rash	6	1.6	0	0
Retained products of conception	19	5.0	3	6.5
Sepsis	9	2.4	5	10.9
Urinary issues	37	9.7	3	6.5
Vaginal bleeding	13	3.4	1	2.2
Wound issues	48	12.6	2	4.3
Total	381	100.0	46	100.0

## Discussion

Our study showed that 5% of new mothers presented to the ED within 10 days of delivery. While other studies have looked at postpartum visits to the ED, they extend the duration of visit up to a year [4,5]. This is the first study to look at the first ten days postpartum and characterize the reasons for presentation as well as identify predictors of admission. The early postpartum period is important as it is an overwhelming time to the new mother. By identifying modifiable factors to prevent early ED visits can be helpful and this was the purpose of our study. Over three quarters of the patients presented within eight days of delivery and the most common complaints were pain and wound-related issues. Only 7.3% and 4.1% of patients in these categories respectively were admitted which would indicate that these visits could be mitigated with appropriate instructions and discharge planning.

We found that patients who were born on a weekend were at increased risk for being admitted during the ED visit. This was not surprising given the reduced complement of healthcare staff such as social workers and lactation consultants over the weekend. This may contribute to a lack of education related to pain management, lactation issues, and discharge instructions. Furthermore, patients who were positive for Group B Strep status (GBS) were less likely to be admitted upon presentation to the ED. With appropriate screening and peripartum prophylaxis, the incidence of maternal infections is decreased [6]. Hence the antibiotics used to treat GBS may confer protection against infections.

Our study was limited due to its retrospective nature conducted at a single health care system. The follow up visits were also only for our ED and we may not have captured women who sought care elsewhere. Furthermore, we excluded patients with a prolonged hospital stay which may have resulted in the exclusion of patients at a higher risk of early ED visits. In addition, we were unable to gather other patient characteristics such as level of education or socioeconomic status, which could be an important contributing factors for the ED visit.

Our study reveals that the majority of postpartum patients that present to the ED can be discharged home. Appropriate discharge instructions pertaining to pain control and wound care would be beneficial as these are the two most common reasons for ED visits. The Enhanced Recovery After Surgery guidelines recommend that patients should be discharged with standardized written discharge instructions, which has also been shown to improve patient satisfaction and recall of instructions [7,8]. Further, the current postpartum visit of 6 weeks may need to be re-examined. The American College of Obstetricians and Gynecologists recommends that all women should be in contact with their obstetrical care provider within the first three weeks as this is the critical period to set the stage of long-term health and wellness [9]. As the majority of patients in our study presented between day 4 and 8, an additional appointment at 1 week will provide an opportunity for patients to discuss their concerns with their provider.

## Conclusion

This study investigated patients who present to the ED in the early postpartum period. By identifying the common reasons for the visit and recognizing that many patients are discharged home, a clinic setting (either Family Medicine or Obstetrics) may be the more appropriate setting to address these patient concerns. Further studies are needed to identify additional predictors of admission.
